# Prolonged control of replication-competent dual- tropic human immunodeficiency virus-1 following cessation of highly active antiretroviral therapy

**DOI:** 10.1186/1742-4690-8-97

**Published:** 2011-12-05

**Authors:** Maria Salgado, S Alireza Rabi, Karen A O'Connell, Robert W Buckheit III, Justin R Bailey, Amina A Chaudhry, Autumn R Breaud, Mark A Marzinke, William Clarke, Joseph B Margolick, Robert F Siliciano, Joel N Blankson

**Affiliations:** 1 Department of Medicine, Johns Hopkins University School of Medicine, Baltimore, Maryland 21287, USA; 2 Department of Pathology, Johns Hopkins University School of Medicine, Baltimore, Maryland 21287, USA; 3 Department of Molecular Microbiology and Immunology, Johns Hopkins Bloomberg School of Public Health, Baltimore, Maryland 21287, USA; 4 Howard Hughes Medical Institute, Johns Hopkins University School of Medicine, Baltimore, Maryland 21287, USA

**Keywords:** HIV-1, elite suppressor, elite controller, viral replication

## Abstract

**Background:**

While initiation of highly active antiretroviral therapy (HAART) during primary HIV-1 infection occasionally results in transient control of viral replication after treatment interruption, the vast majority of patients eventually experience a rebound in plasma viremia.

**Results:**

Here we report a case of a patient who was started on HAART during symptomatic primary infection and who has subsequently maintained viral loads of < 50 copies/mL for more than nine years after the cessation of treatment. This patient had a high baseline viral load and has maintained a relatively high frequency of latently infected CD4^+ ^T cells. In addition, he does not have any known protective HLA alleles. Thus it is unlikely that he was destined to become a natural elite controller or suppressor. The mechanism of control of viral replication is unclear; he is infected with a CCR5/CXCR4 dual-tropic virus that is fully replication-competent *in vitro*. In addition, his spouse, who transmitted the virus to him, developed AIDS. The patient's CD4^+ ^T cells are fully susceptible to HIV-1 infection, and he has low titers of neutralizing antibodies to heterologous and autologous HIV-1 isolates. Furthermore, his CD8^+ ^T cells do not have potent HIV suppressive activity.

**Conclusion:**

This report suggests that some patients may be capable of controlling pathogenic HIV-1 isolates for extended periods of time after the cessation of HAART through a mechanism that is distinct from the potent cytotoxic T lymphocyte (CTL) mediated suppression that has been reported in many elite suppressors.

## Background

HIV-1 infection results in extensive viral replication and progressive CD4^+ ^T cell depletion in the vast majority of patients. However, rare subjects, known as elite controllers or suppressors (ES), spontaneously control viral replication without antiretroviral treatment [1]. The mechanisms involved in elite control are not fully understood, but some ES appear to be infected with fully replication-competent virus [2-5] that continues to evolve during chronic infection [6-8]. Thus infection with attenuated virus does not appear to be a common cause of elite control. In contrast, many studies looking at host factors have shown that the HLA-B*27 and 57 alleles are overrepresented in ES [9-14]. This has strongly suggested a role for CD8^+ ^T cell responses in elite control, and indeed, potent HIV-specific CD8^+ ^T cell responses [15-17] that are capable of inhibiting viral replication [18,19] have been documented in many ES.

It is not clear whether it will be possible to elicit similar levels of immune control in patients with progressive HIV-1 disease. However, some studies have suggested that rare individuals who are treated early in primary infection with highly active antiretroviral therapy (HAART) are able to control viral replication when therapy is discontinued. Rosenberg and colleagues demonstrated that five of eight patients who were treated before or shortly after seroconversion were able to suppress HIV RNA levels to below 500 copies/mL for a median of 6.5 months after therapy was interrupted [20]. However, a follow up study showed that this control was of limited duration as only three of 14 patients who started HAART during primary infection maintained viral loads of < 5000 copies/mL two years after treatment interruption [21]. In another study, a patient who was started on HAART a month after seroconversion was treated for four years prior to a treatment interruption which resulted in a rapid rebound in viremia. HAART was reinitiated and ultra-low doses of interleukin-2 (1.2 mIU/m^2^/day) were added to the regimen. Interestingly, he maintained viral loads of < 50 copies/mL for 14 months after both HAART and IL-2 were discontinued [22]. In a recent study, five of thirty-two patients treated during primary HIV-1 infection maintained control of viral replication for more than six months after treatment was interrupted [23]. While this phenomenon is not routinely seen with early treatment [24-26], these cases strongly suggest that the immune system can be manipulated to control HIV-1 replication in some patients. Thus, this could be the basis for the design of a successful therapeutic vaccine.

We present a case of a patient infected with a replication-competent, dual-tropic HIV-1 isolate who was started on treatment during primary infection. He has maintained stable CD4+ T cell counts and viral loads of < 50 copies/ml for more than nine years since HAART was discontinued. To our knowledge, this represents the longest period of control of HIV-1 replication in a patient after the cessation of treatment. We performed detailed analyses of the patient's viral isolates and looked at multiple aspects of his HIV-specific immune response. While no clear mechanism of immune control was identified, this case suggests that long term control of pathogenic HIV-1 isolates is possible in some patients who were destined to become chronic progressors (CP).

## Results

### Patients

**Figure 1:**
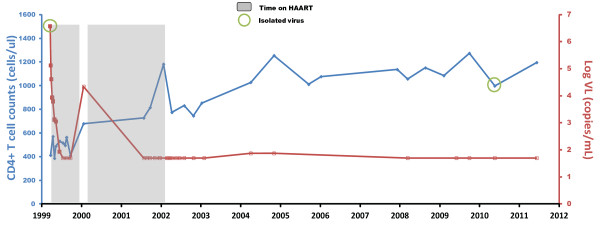
**Clinical characteristics of Patient 169**. The patient's CD4 counts and viral load are shown. Viral load measurements below the limit of detection are denoted by open symbols. The time on HAART is denoted by the shaded region.

Patient 169 is a 57 year old male who was diagnosed with primary HIV-1 infection when he admitted to the intensive care unit at Johns Hopkins Hospital in 1999 with severe HIV-1 meningoencephalitis that resulted in intubation for airway protection [27]. He was found to have an indeterminate Western blot (only bands to p24 were present) and an HIV-1 viral load of > 750,000 copies/mL. He reported having tested negative for HIV-1 two years prior to admission. He was enrolled into the Acute Infection and Early Disease Research Program (AIEDRP) study and started on a regimen of zidovudine, lamivudine, and indinavir within 48 hours of admission. This regimen was changed to abacavir, lamivudine and efavirenz at week four and by week 16, his viral load was < 50 copies/ml. He stopped taking his antiretrovirals at week 36 for a 2 week period, and his viral load rebounded to 22,000 copies/ml. The same regimen was re-initiated, and he was adherent until week 92 at which time he stopped taking all of the antiretroviral drugs. His CD4^+ ^T cell count, which was 412 cell/μL at the time of diagnosis, has been stable at greater than 1000 cells/μL over the last five years, and his viral load, which has been consistently less than 50 copies/ml since the discontinuation of HAART, was measured at 1 copy/mL in 2011 using a highly sensitive single copy assay [28,29] (Figure 1). The patient was incarcerated between 2004 and 2005, and medical records confirmed that he was not on antiretroviral therapy at this point. Furthermore, qualitative testing for antiretroviral drugs on plasma samples from 2009, 2010 and 2011 was performed to rule out surreptitious use of antiretroviral therapy. All samples were negative whereas nevirapine and lamivudine were detected in a plasma sample from his spouse.

The patient's spouse was diagnosed with HIV-1 infection 3 years before subject 169 was admitted with acute retroviral syndrome. Her CD4^+ ^T cell count nadir was 84 cells/μL, and her baseline viral load prior to the initiation of HAART was 122,000 copies/ml.

### Patient 169 has a high frequency of HIV-1 infected CD4^+ ^T cells

In order to determine whether the patient was infected with a defective virus and whether his spouse transmitted the virus to him, we amplified virus from a plasma sample from the time of diagnosis. In addition, virus was cultured from CD4^+ ^T cells isolated from PBMCs obtained from the patient and his spouse in 2010. The frequency of latently infected resting CD4^+ ^T cells in patient 169 was 1.61 infectious units per million, which is more than a log higher than the frequency found in our cohort of ES [3] and similar to the frequencies found in chronic progressors on suppressive HAART regimens [30,31].

### Patient 169 is infected with fully replication-competent, dual-tropic virus

**Figure 2:**
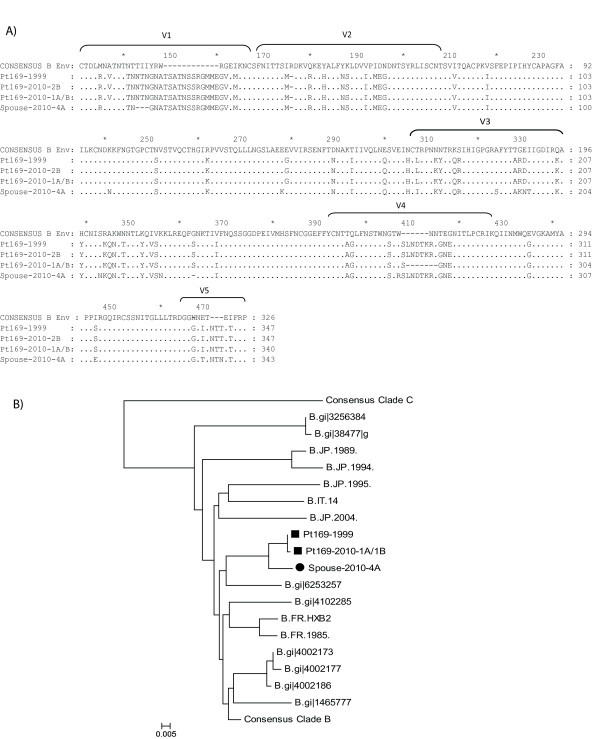
**Phylogenetic Analysis: An alignment of the variable regions of env is shown for replication-competent isolates obtained from Patient 169 and his spouse**. Numbering is from the first amino acid in gp120. (A). The sequences are also compared to other Clade B sequences(B). Phylogenies were estimated by using a classical approach, functioning under a maximum-likelihood (ML) optimality criterion.

We next analyzed the fitness of isolates obtained from patient 169 and his spouse. For patient 169, full genome sequencing of replication-competent virus cultured from 1999 plasma and three independent replication-competent isolates obtained from CD4^+ ^T cells in 2010 was performed. One of the isolates from 2010 (2B) was identical to the 1999 isolate with the exception of a single nucleotide difference in the HIV-1 LTR. The two other isolates from 2010 (1A, 1B) were identical although they were isolated from independent culture wells. The differences between the identical 2010 isolates and the 1999 isolate are summarized in Table 1. For the patient's spouse, full genome sequencing of two independent isolates cultured from her resting CD4^+ ^T cells in 2010 was performed. No large deletions were found in any gene and no drug resistance mutations were found in any of the isolates obtained from either patient. Phylogenetic analysis of the *env *gene showed that the isolates from 169 and his spouse were more closely related to each other than to any other isolate in the Los Alamos database, confirming that the two patients were a transmission pair [Figure 2].

**Table 1:** Differences in sequence of replication-competent 1999 and 2010 isolates.

	Differences between Pt-169 1999 and 2010-1A/1B isolates	
	
	Nucleotides	Amino Acids
LTR	2	

Gag	Δ18*	Δ6*

Pol	2	2

Vif	0	0

Vpr	1	1

Vpu	0	0

Env	2, Δ21 (V4)*	1, Δ7 (V4)*

Nef	1	1

Total	47	18

Isolate 2B from 2010 is identical to the 1999 isolate with the exception of a single nucleotide in the LTR. Isolates 1A and 1B are identical to each other.

* The triangle denotes deletions in the 2010-1A/1B isolates.

**Figure 3:**
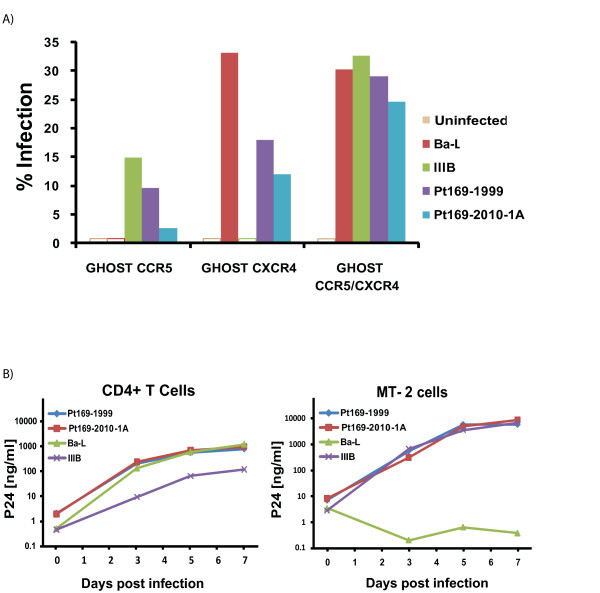
**Phenotypic analyses of viral isolates from Patient 169**. (A) Viral tropism was determined using GHOST cells expressing CCR5 and/or CXCR4. Open bars were below the limit of the detection (B) Replication kinetics was determined in primary CD4+ T cells (left) and the MT-2 cell line (right).

Sequence analysis of the *env *gene suggested that all isolates cultured from both patients were CXCR4- tropic (data not shown). To confirm this, we amplified and cloned the *env *gene from the 1999 and 2010 isolates from patient 169, and made GFP-expressing NL4-3 pseudotyped virus as previously described [32]. Infection studies were then performed with GHOST cells expressing CCR5 and/or CXCR4. As shown in Figure 3A, pseudotyped virus containing *env *from 1999 and 2010 was able to infect GHOST cells expressing either co-receptor, demonstrating that each viral clone was dual-tropic (Figure 3A).

We next compared the replication capacity of virus cultured from patient 169 to that of CCR5-tropic (Ba-L) and CXCR4-tropic (IIIB) laboratory isolates. As shown in Figure 3B, the isolates from 1999 and 2010 replicated as well as IIIB in MT-2 cells whereas Ba-L did not replicate in these cells, which do not express the CCR5 co-receptor. In primary CD4^+ ^T cells, the two isolates from Patient 169 replicated as well as Ba-L. Thus control of viral replication in this patient was not due to infection with an attenuated virus, and viral fitness was stable over time.

### Patient 169 does not have known genetic factors that contribute to the control of viral replication

Having ruled out viral attenuation, we focused on host factors as potential causes of the observed virologic control. Heterozygosity for the 32 base pair deletion in CCR5 has been associated with slow HIV-1 progression [33,34]. This gene was thus analyzed by PCR, and patient 169 was determined to have two wild type CCR5 alleles. The most consistent finding in different cohorts of ES has been the over-representation of protective HLA alleles such as HLA-B*27 and B*57 [9-14,35,36]. In addition, genome wide association studies have documented a protective single nucleotide polymorphism (SNP) in the HLA-C promoter [35,36]. Furthermore, HLA-Bw4-80Ile alleles have been shown to be associated with slowly progressive disease when inherited in conjunction with the KIR3DS1 and/or KIR3DL1 natural killer cell receptor alleles [37,38]. Patient 169 does not have an HLA-Bw4-80Ile allele or any other HLA allele that has been previously associated with attenuated HIV-1 disease. He also does not have the protective C/C HLA-C SNP (Table 2).

**Table 2:** Analysis of genetic factors associated with protection in HIV-1 infection

Genetic factors	
**Factor**	**Genotype**

CCR5	wild type

HLA-A HLA-B HLA-C	*3001, *6801 *4201 *0602, *1701

HLA C SNP (rs9264942)	T/C

### CD4^+ ^T cells from Patient 169 are fully susceptible to infection

**Figure 4:**
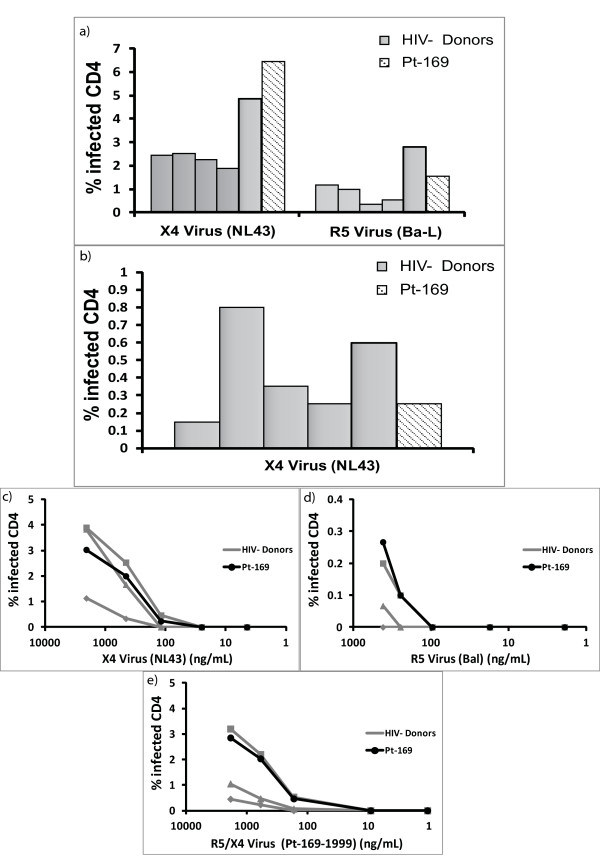
**CD4+ T cell susceptibility assay**. CD4+ T cells from five healthy donors (grey columns) and Patient 169 (shaded column) were infected with CCR5 tropic (Bal) and CXCR4 tropic (NL43) pseudotypevirus by spinoculation (A) or with NL43pseudotype virus without spinoculation (B). Infection with serial dilutions of CXCR4-tropic (C), CCR5-tropic (D) and dual-tropic virus (E) was also performed by spinoculation. The percent of infected cells (GFP positive) are shown.

Some studies [39,40] have suggested that ES CD4^+ ^T cells that have been activated *ex vivo *are resistant to viral infection while others have shown that unstimulated CD4^+ ^T cells from these patients are fully susceptible to viral entry and productive infection [41,42]. In order to determine whether CD4^+ ^T cells from patient 169 were resistant to infection, we purified primary CD4^+ ^T cells from 169 and five HIV-1 seronegative donors and infected them directly *ex vivo *by spinoculation with CCR5 (Ba-L) and CXCR4 (NL4-3) tropic isolates as previously described. As shown in Figure 4, CD4^+ ^T cells from 169 were as susceptible to infection with both types of isolates as were the CD4^+ ^T cells from the seronegative donors. In order to determine if spinoculation was masking subtle differences in the susceptibility to infection, we infected CD4^+ ^T cells with CXCR4-tropic virus without spinoculation [41] and again found that CD4^+ ^T cells from patient 169 were fully susceptible to infection. We also looked at susceptibility to infection with serial dilutions of both lab strains and a pseudotyped virus containing dual tropic envelope that was amplified from the patient in 1999. In all cases, patient 169's cells were found to be as susceptible to infection as cells from HIV-negative donors.

### Patient 169 has low titers of HIV-specific neutralizing antibodies

**Figure 5:**
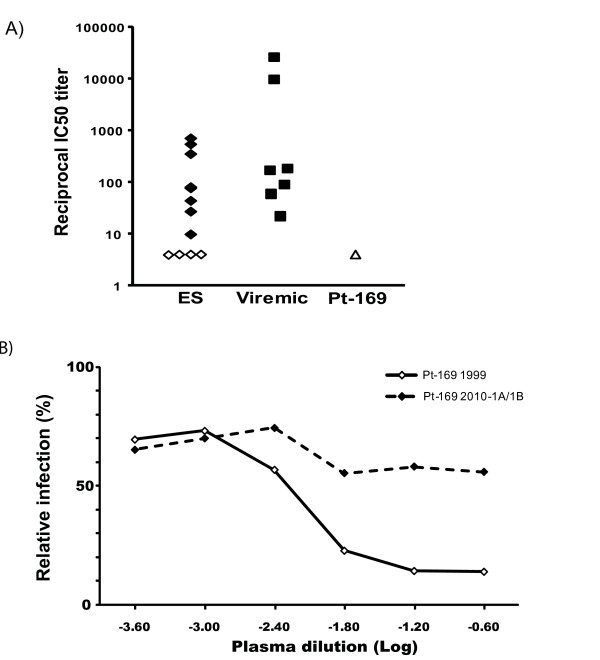
**Titers of HIV-specific neutralizing antibodies**. (A) Neutralizing activity of plasma from viremic patients, ES, and patient 169 against recombinant virus with Env from the laboratory strain SF162. The open symbols denote titers that were greater than 1:4. (B) Neutralizing activity of plasma from Patient 169 against recombinant virus with Env from 1999 and 2010-1A isolates. Relative infection of TZMb1 cells by the recombinant isolates is shown in the presence of four fold dilutions of plasma.

To determine whether a robust humoral response was playing a role in the control of viral replication, we compared reciprocal IC50 titers of neutralizing antibodies (Nab) in patient 169 to titers in viremic patients and ES as previously described [43]. Patient 169 had the lowest titers of Nab to laboratory strain SF162 Env as shown in Figure 5. To determine how well the patient neutralized autologous virus, we measured titers of Nab to pseudotyped virus expressing Env cloned from the 1999 and 2010 replication-competent isolates. His reciprocal IC 50 titers of Nab to autologous Env from 1999 was 1:195 which is comparable to the titers seen in ES, but lower than the titers seen in viremic patients [43]. In contrast, his Nab titer to contemporaneous Env was >1: 4 (Figure 5B). Thus it appears that neutralizing antibodies were not the cause of control of viral replication in this patient.

### Characteristics of the HIV-specific CD8^+ ^T cell response in Patient 169

**Figure 6:**
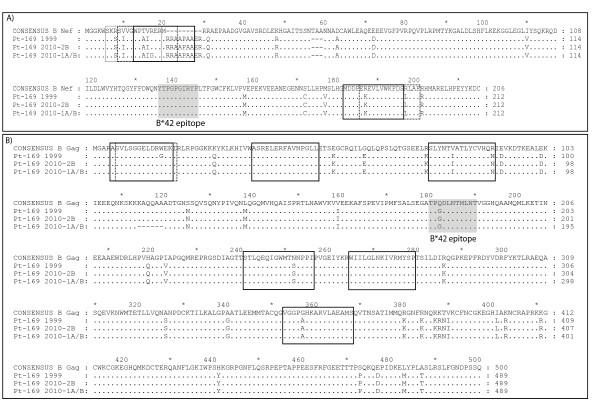
**CD8+ T cell epitope analysis**. Epitopes in Nef (A) and Gag (B) targeted by CD8+ T cells as determined by an IFN-g ELISPOT assay using overlapping 15 mers. Open boxes represent actual peptides targeted in the assay whereas the shaded boxes represent predicted optimal HLA-B*42 restricted epiotpes which were not targeted.

Many ES have been found to possess potent HIV-specific CD8^+ ^T cell activity [16-19]. We thus looked at CD8^+ ^T cell responses in Patient 169. An ELISPOT assay was performed following stimulation with Gag and Nef peptides. As shown in Figure 6, two independent non-overlapping epitopes were targeted in Nef, and and six such epitopes were targeted in Gag. Escape mutations in certain Gag epitopes have been associated with viral attenuation [44-46], and we therefore examined sequences in targeted epitopes to look for signs of virologic escape. A comparison of the sequences from the 1999 and 2010 isolates showed an R18G substitution in a Nef epitope and a G17W substitution in a Gag epitope in isolates 1A and 1B. Both substitutions were absent in isolate 2B, and thus even if these mutations caused a reduction in viral fitness in some isolates, escape mutations would not explain virologic control in this patient.

**Figure 7:**
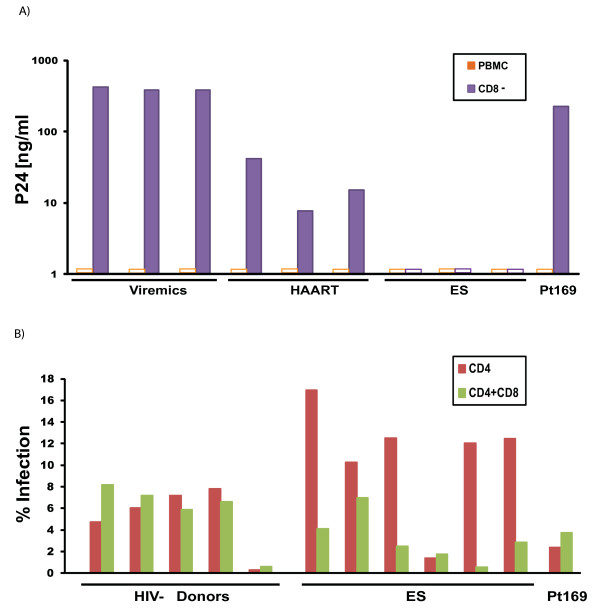
**CD8+ T cell functional analysis**. The effect of CD8+ T cell depletion on autologous virus outgrowth is shown by a comparison of virus replication in activated unfractionated PBMC or activated PBMC from which CD8+ T cells were depleted (A). The ability of CD8+ T cells to inhibit replication of GFP expression virus in activated autologous CD4+ T cells is shown. Open bars were below the limit of the detection. (B) by comparing the percentage of infected CD4+ T cells (GFP positive) in the absence and presence of CD8+ T cells.

To determine whether CD8^+ ^T cells were involved in the direct control of viral replication, we attempted to culture autologous virus from Patient 169 with and without the depletion of CD8^+ ^T cells. As shown in Figure 7A, virus culture was successful only when CD8^+ ^T cells were depleted, but the same phenomenon was seen in chronic progressors who had substantial levels of viremia and in patients on suppressive HAART regimens. There was no viral outgrowth from ES CD4^+ ^T cells consistent with the low frequency of infected CD4^+ ^T cells in these patients [3]. We next compared the respective abilities of CD8^+ ^T cells from Patient 169 and ES in HIV-1 inhibition assays in which pseudotyped virus was used to superinfect autologous CD4^+ ^T cells. As shown in Figure 7B, while primary CD8^+ ^T cells from most ES caused a significant reduction in virus replication, CD8^+ ^T cells from Patient 169 had very little effect in this assay.

## Discussion

We present here a patient who has controlled HIV-1 replication for more than nine years after the cessation of HAART. While some studies have reported that initiation of HAART during primary infection can lead to the control of viral replication once therapy is discontinued, most of these patients eventually experienced a rebound in viremia [21,22]. To our knowledge, the nine years of control seen in patient 169 is the longest period of control reported in a patient who was treated in primary infection and who subsequently underwent treatment interruption. We extend prior studies by performing full genome sequence analysis and phenotypic studies of viral isolates obtained at the time of infection and eight years after the cessation of HAART. We show that Patient 169 was infected by virus from a patient with AIDS, and we demonstrate that the viral isolates from patient 169 are dual-tropic and replication-competent, which makes it unlikely that an attenuated virus was transmitted. It should be noted that infection with dual-tropic virus is associated with more rapid progression than infection with CCR5-tropic virus [47,48]. Thus the long term control seen in this patient is even more remarkable.

We hypothesized that the patient's virus may have developed drug resistance mutations or escape mutations that led to viral attenuation later in his disease course. However sequence analysis did not reveal any drug resistance mutations, and potential escape mutations in Gag and Nef were seen in only some isolates. While it is possible that escape mutations in other viral genes caused a reduction in viral fitness, the fact that isolates obtained from 2010 replicated as well in vitro as viral isolates present during primary infection makes this unlikely.

We considered the possibility that this patient was destined to become a natural ES. However several observations suggest that this is not the case. He had a viral load of > 750,000 copies/mL and was very symptomatic during seroconversion. Studies have shown that patients with severe acute retroviral syndrome have a more rapid rate of disease progression [49]. In contrast, natural ES tend to have limited symptoms and low viral loads during primary infection [50-52]. ES also invariably have extremely low frequencies of latently infected CD4^+ ^T cells [3] whereas Patient 169 had a very large number of HIV-1 infected CD4^+ ^T cells during primary infection [27], and his current frequency of latently infected cells is currently similar to that seen in patients with progressive disease on HAART. Finally, he did not have any of the HLA alleles that are overrepresented in ES.

We show here that CD4^+ ^T cells from Patient 169 are fully susceptible to infection and that he had very low titers of neutralizing antibodies to heterologous and autologous virus. Interestingly, depletion of CD8^+ ^T cells resulted in efficient outgrowth of virus from CD4^+ ^T cells. While this suggests that CD8^+ ^T cells are playing a role in the control of viral replication, it is unlikely to be the only mechanism involved as CD8^+ ^T cells from patients with progressive disease were also effective at preventing outgrowth of autologous virus. In contrast, CD8^+ ^T cells from Patient 169 were not as effective as those from ES at inhibiting replication of recombinant virus carrying GFP. Thus it appears that this patient is controlling replication of pathogenic dual-tropic virus by a mechanism that is distinct from the CD8^+ ^T cell mediated control that is seen in many ES. This unknown mechanism may be similar to the mechanisms present in ES who do not possess protective HLA alleles or potent HIV-specific CD8^+ ^T cell responses [9,13,53], but it is still unique in that control is being maintained over a much larger number of infected CD4+ T cells in Patient 169.

## Conclusions

The data presented here suggest that early treatment in some patients infected with fully pathogenic virus can lead to control of viral replication for extended periods of time. Understanding the mechanisms involved in this control may lead to vaccine development and effective immunotherapy in patients with progressive disease.

## Availability of supporting data

The data sets supporting the results of this article are available in the Gen Bank repository (accession numbers JN599164 and JN599165)

## Methods

### Virus Isolation and Sequence Analysis

Culture of replication-competent virus from CD4^+ ^T cells was performed as previously described [3]. Replication-competent virus from 1999 was obtained by spinoculating CD4^+ ^T cells from an uninfected donor with the patient's plasma. Full genome sequence analysis of viral isolates was performed as previously described [3]. Classical, maximum likelihood and Bayesian phylogenetic analysis were performed as described previously [7].

### Antiretroviral drug testing

100 μl of patient serum were treated with 300 μl of cold acetonitrile, stored at -20°C for 20 minutes and subsequently centrifuged at 12,000 × rpm for 5 minutes. Specimen supernatants were evaporated to dryness and reconstituted with 100 μl water. 10 uL of each treated sample were injected onto the liquid chromatography system equipped with Transcend pumps (Thermo Fisher Scientific) for analytical separation. The chromatographic run began with 60 seconds of 5% methanol containing 10 mM ammonium acetate (mobile phase B), followed by a 10 minute ramp to 95% B. Analytes were eluted from a Hypersil Gold 50 × 2.1 mm; 3 μm particle size HPLC column (ThermoFisher Scientific) during this gradient and the column was washed for 60 seconds with 2:2:1 acetonitrile:isopropanol:acetone and re-equilibrated with 5% mobile phase B for 180 seconds. Analytes were detected over a 14.9 minute run using the Exactive Orbitrap mass analyzer (Thermo Fisher Scientific) with a heated electrospray ionization (HESI) source. The source parameters were as follows: sheath gas: 40, auxillary gas: 10, sweep gas: 0, spray voltage: 3.5 kV, capillary temperature: 270 °C, capillary voltage: 60 V, tube lens voltage: 120 V, skimmer voltage: 15 V, heater temperature: 350 °C. The mass spectrometer method included two positive-mode scan events: one full scan event with ultra-high resolution (100000 @ 1 Hz) and one in-source collision-induced dissociation (CID) event with enhanced resolution (25000 @ 4 Hz) and collision energy of 45 eV. Both scan events were programmed for 100 ms maximum inject time and balanced ACG targets. The analytical method was found to have a limit of detection of <20 ng/ml for amprenavir, atazanavir, darunavir, efavirenz, emtricitabine, indinavir, lamivudine, lopinavir, nelfinavir, nevirapine, ritonavir, saquinavir, tenofovir and tipranavir. Positive identification was determined by exact mass detection at 5 ppm discrimination, analyte retention time and identification of mass transitions when possible.

### Viral Tropism assay

RFP expressing recombinant pseudotype virus was made with *env *genes amplified from 1999 and 2010 isolates. These viruses were used to infect GHOST cell lines transfected with CCR5 and/or CXCR4 (obtained from the NIH AIDS Research and Reference Program) and the percentage of RFP positive cells was determined in triplicates on day three. GHOST cells express low levels of endogenous CXCR4 and therefore infection of cells transfected with CCR5 alone was performed in the presence of the CXCR4 antagonist AMD 3100 at a dose of 1 uM (obtained from the NIH AIDS Research and Reference Program).

### Viral Fitness Assay

Viral fitness was analyzed as described previously [3]. PBMCs from healthy donors were activated for two days with IL-2 and PHA. CD4^+ ^T cells were isolated (MACS, CD4^+ ^T cell isolation Kit) and infected by spinoculation [54] (1200 × g for 2 hours) with equal quantities (200 ng/mL) of p24 from primary patient isolates, or with Ba-L or IIIB laboratory HIV-1 strains as controls. Supernatant samples were taken over the course of 7 days. Viral replication was quantified using p24 ELISA (Perkin Elmer).

### Genetic Polymorphisms

HLA-A, B, and C allele identification was performed at the Johns Hopkins University Immunonogenetics laboratory. CCR5 was amplified from genomic DNA using gene specific primers. The presence or absence of the CCR5 Δ32 mutation was determined by relative size of the resulting PCR fragment. HLA-C single nucleotide polymorphism genotyping (rs9264942) was performed utilizing the Applied Biosystems 7300 real-time PCR System allelic discrimination assay, following the manufacturer's guidelines. Primers and probes were developed by Custom TaqMan SNP Genotyping assays (ABI). Determination of the HLA-B Bw4-80Ile allele was performed using the Olerup SSP 104.101 KIR Genotyping 12 Lot71E and 104.201 KIR ligand genotyping Lot85E kits, following the manufacturer's guidelines.

### CD4^+ ^T cell susceptibility assay

CD4^+ ^T cells from the patient and five healthy donors were purified by negative selection using Miltenyi beads and were infected directly ex vivo. Spinoculation [55] was performed with pseudotyped CCR5 and CXCR4 tropic viruses and GFP expression was measured in triplicates as previously described [41,42]. Infection without spinoculation was also performed with CXCR4 tropic virus.

### Neutralization assay

Neutralization assays were performed as described previously [43]. Briefly, recombinant pseudoviruses containing SF162, or Patient 169 *env *were titrated on TZM b1 cells to determine a linear range of infection for each pseudovirus. Infections were then performed in duplicate with a concentration of virus within this linear range, along with serial dilutions of patient plasma that had been heat inactivated at 56°C for 60 min. All assays were performed in the presence of 10% total human plasma. Each virus was pre-incubated with 5% test plasma and with four-fold serial dilutions of test plasma in normal human plasma. To determine neutralization, each test plasma well was compared to wells containing an equal concentration of normal human plasma.

### CD8^+ ^T cell assays

Reactive CTL epitopes were defined by IFN-γ Elispot. As previously described [55], whole blood was taken from each patient and PBMCs were isolated by Ficoll gradient centrifugation. PBMCs were aliquoted into each well of 96 well MultiScreen (Millipore) plates with conjugated IFN-γ antibody. Cells were activated with overlapping peptides spanning the entire amino acid sequence of B clade consensus *gag *and *nef *at a concentration of 5 μg/ml (obtained from the NIH AIDS Research and Reference Program). PBMCs were cultured overnight, and subsequently analyzed. Quantification of spot forming units (SFU) was performed in a blinded fashion by Zellnet Consulting (Fort Lee, NJ). Positive responses were defined as greater than 50 SFU per million PBMCs. Negative controls (wells with medium alone) routinely had less than 15 SFU per million PBMC.

The effect of CD8+ T cells on autologous virus outgrowth was determined by measuring p24 values on unfractionated PBMC and PBMC depleted of CD8+ T cells. The cells were stimulated for 48 hours with PHA at 1 μg/ml and culture supernatant was obtained on day 10.

The cytolytic T cell effect was determined by a CD8 suppression assay. PBMCs were isolated from patients, and CD8^+ ^T cells were positively selected using Miltenyi magnetic beads (MACS, CD8^+ ^T cell Isolation kit). CD8^+ ^T cells were depleted of CD16^+ ^cells (Invitrogen, Dynal Beads) to remove contaminating NK cells. CD4^+ ^T cells were isolated by negative selection using Miltenyi magnetic beads. Purity of depletion was analyzed by flow cytometry. CD4^+ ^T cells were infected by spinoculation at 1200 × g for two hours with replication competent NL4-3 virus, in which GFP is engineered into *nef*. Flow cytometry was performed five days after infection to assess the percentage of GFP positive cells.

## Competing interests

The authors declare that they have no competing interests.

## Authors' contributions

MS, SAR, KOC, and RWB performed all the experiments and helped draft the manuscript. JRB helped to design the neutralization antibody assay. ARB, MAM, and WC tested plasma samples for antiretroviral drugs. AAC and JBM provided clinical samples and helped draft the manuscript. RFS participated in the study design and helped to draft the manuscript. JNB conceived of the study, participated in its design and coordination and helped to draft the manuscript. All authors read and approved the final manuscript.
